# Multi-Scale Adaptive Light Stripe Center Extraction for Line-Structured Light Vision Based Online Wheelset Measurement

**DOI:** 10.3390/s26020600

**Published:** 2026-01-15

**Authors:** Saisai Liu, Qixin He, Wenjie Fu, Boshi Du, Qibo Feng

**Affiliations:** 1 School of Physical Science and Engineering, Beijing Jiaotong University, Beijing 100044, China; 21111106@bjtu.edu.cn (S.L.); qbfeng@bjtu.edu.cn (Q.F.); 2 MOE Key Laboratory of Luminescence and Optical Information, Beijing Jiaotong University, Beijing 100044, China

**Keywords:** centerline extraction, line structured light, parameter adaptation, vision measurement

## Abstract

The extraction of the light stripe center is a pivotal step in line-structured light vision measurement. This paper addresses a key challenge in the online measurement of train wheel treads, where the diverse and complex profile characteristics of the tread surface lead to uneven gray-level distribution and varying width features in the stripe image, ultimately degrading the accuracy of center extraction. To solve this problem, a region-adaptive multiscale method for light stripe center extraction is proposed. First, potential light stripe regions are identified and enhanced based on the gray-gradient features of the image, enabling precise segmentation. Subsequently, by normalizing the feature responses under Gaussian kernels with different scales, the locally optimal scale parameter (*σ*) is determined adaptively for each stripe region. Sub-pixel center extraction is then performed using the Hessian matrix corresponding to this optimal *σ*. Experimental results demonstrate that under on-site conditions featuring uneven wheel surface reflectivity, the proposed method can reliably extract light stripe centers with high stability. It achieves a repeatability of 0.10 mm, with mean measurement errors of 0.12 mm for flange height and 0.10 mm for flange thickness, thereby enhancing both stability and accuracy in industrial measurement environments. The repeatability and reproducibility of the method were further validated through repeated testing of multiple wheels.

## 1. Introduction

Machine vision-based technology has been extensively employed in dimensional metrology systems. In this technology, object-related information is captured via image sensors, with key features extracted through edge detection, deep learning, and other relevant techniques, enabling dimensional measurement and surface defect identification [1,2,3]. As a specialized branch of machine vision, line-structured light vision sensing technology been widely embraced in train wheelset profile measurement owing to its inherent merits of non-contact operation, high precision, and reliable sensing performance [4,5,6]. In such a system, a line-structured light pattern is projected onto the wheel tread surface by a laser, and the stripe image containing profile information is captured by an industrial camera. The tread profile is then reconstructed through light stripe center extraction and coordinate transformation [7,8]. The accuracy of light stripe center extraction directly influences the accuracy of the measurement process. Therefore, numerous studies on light stripe center extraction have been carried out in recent years, and the relevant methods are primarily categorized into geometric center methods and energy center methods [9,10,11]. Geometric center methods typically include the threshold method [12], extreme value method [13], edge method [14], refinement extraction [15], and improvements based on these approaches [16]. These methods are based on the assumption that the light stripe center coincides with its geometric center, offering straightforward implementation and high computational efficiency. However, they often ignore the actual gray intensity distribution of the light stripe on the measured surface. Energy center methods include the gray centroid method [9,17], curve fitting [18], directional template methods [19], and algorithms based on the Hessian matrix [20,21,22]. These methods characterize the light stripe’s intensity distribution based on its gray-level distribution, treating the stripe center as the intensity peak. Such approaches generally exhibit greater stability against interference and higher accuracy. Over the past few years, the Hessian matrix, which characterizes the gray-level gradient distribution in images, has been extensively utilized by researchers worldwide for light stripe center extraction and image segmentation [23,24,25]. As a key parameter for Hessian matrix computation, the standard deviation (*σ*) of the Gaussian convolution kernel influences the accuracy and stability of light stripe center extraction by modulating the degree of image smoothing [26,27,28]. Traditional Steger methods employ a Gaussian kernel with a fixed *σ* value, which fails to adapt to images with significant variations in stripe width, leading to reduced center extraction accuracy and measurement errors. Zhao et al. [29] employed regional segmentation to process complex patterns, applied tailored convolution operations to different regions to enhance stripe quality, and extracted the stripe center by operating on specific areas with corresponding convolution kernels. Li et al. [30] proposed a hardware-oriented algorithm that estimates the local stripe width and optimal Gaussian kernel size via pixel traversal for center extraction. To address the challenge of light stripe center extraction on highly reflective surfaces, Bo et al. [26] proposed using a BPNN (Back Propagation Neural Network) to estimate local stripe widths and build an adapted Hessian matrix for precise sub-pixel center calculation. Peng et al. [31] employed a modified YOLOv8 network to segment light stripes and estimate their widths. Subsequently, multi-scale Gaussian templates were constructed based on the estimated widths to achieve sub-pixel center extraction. Jiang et al. [32] adopted a U-Net architecture to segment light stripes from complex backgrounds, followed by the application of the Steger algorithm for precise sub-pixel center extraction. However, current methods have not yet achieved an optimal balance among adaptability, efficiency, and practicality: traditional approaches struggle to flexibly handle varying stripe widths and measured surfaces with complex, rich features, while deep learning–based methods are constrained by their reliance on large datasets, substantial computational resources, and system complexity. Therefore, a universal and adaptive approach for light stripe center extraction that is independent of extensive training data retains substantial research value and industrial significance.

To address the challenges of low accuracy and stability in extracting stripe centerlines caused by varying stripe widths and non-uniform grayscale distributions in industrial applications, a multi-scale adaptive center extraction method is proposed to improve the accuracy and stability of center extraction by achieving scale matching between Gaussian kernel parameters *σ* and stripe width *w*. First, the feature responses of the light stripe at multiple *σ* scales are calculated to enhance stripe features and achieve stripe segmentation. Then, by adaptively selecting the optimal *σ* from normalized multi-scale eigenvalues, a scale parameter best matching the local stripe width is determined for center extraction, enabling sub-pixel coordinate acquisition. By integrating multi-scale light stripe gradient features, this method effectively addresses the issues of uneven gray-level distribution and varying stripe widths in the image, exhibiting high stability and accuracy.

## 2. Light Stripe Image Acquisition and Analysis

### 2.1. Measurement System Structure

**Figure 1:**
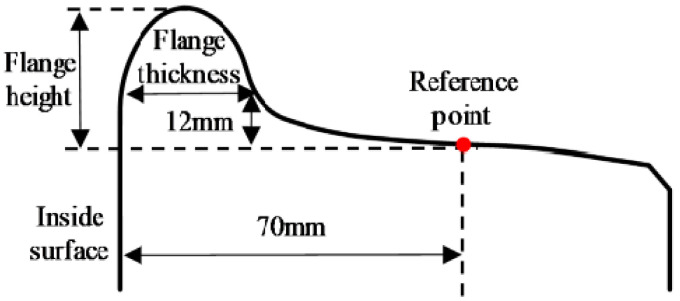
Definition of wheel profile geometric parameters.

The geometric parameters of train wheel profiles mainly include flange height and flange thickness. The LM-type wheel profile commonly used in China, along with its parameter definitions, is illustrated in Figure 1. At a distance of 70 mm from the inner side of the wheel, a point is designated as the reference point. The vertical distance from the reference point to the flange top is defined as the flange height. The horizontal width of the flange, measured 12 mm above the reference point, is defined as the flange thickness.

**Figure 2:**
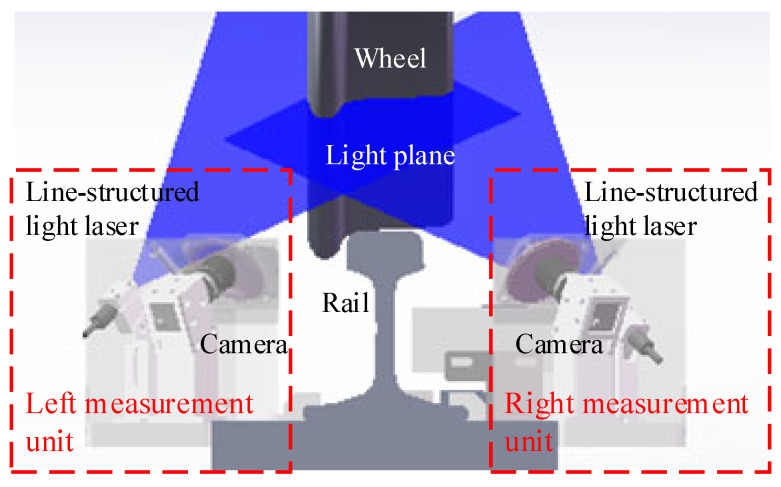
Schematic diagram of the wheel profile measurement system.

A wheel profile measurement system was designed to achieve online non-contact detection of the tread profile, as illustrated in Figure 2. The system comprises left and right measurement units installed symmetrically along the rail. Each unit integrates a line-structured-light laser and an industrial camera, forming a vision sensor. Two sets of line-structured-light lasers project structured light onto the wheel tread from both sides of the rail, covering the complete profile. Cameras on each side capture images of the profile light stripes from specific angles. The center coordinates of the stripes are extracted from the images of both sides and are then converted into real-world profile coordinates using pre-calibrated camera parameters. Finally, key profile parameters are calculated according to their defined specifications.

### 2.2. Light Stripe Feature Analysis

An ideal line-structured light stripe exhibits varying widths without significant distortion or fluctuation, smooth and continuous gray-level transitions with high contrast against the background, and the distribution of normal light intensity is usually approximately a Gaussian distribution [18,33]. However, due to contact wear between the wheel and rail, the surface roughness of the wheel tread varies by region, leading to non-uniform reflectivity. This variability poses a major challenge for accurate measurement using laser vision sensors, as it induces stripe width variations and uneven gray-level distribution, thereby degrading the quality of acquired stripe images. Such degradation interferes with subsequent light stripe segmentation and center extraction, ultimately reducing the accuracy of both center extraction and profile detection [34,35].

**Figure 3:**
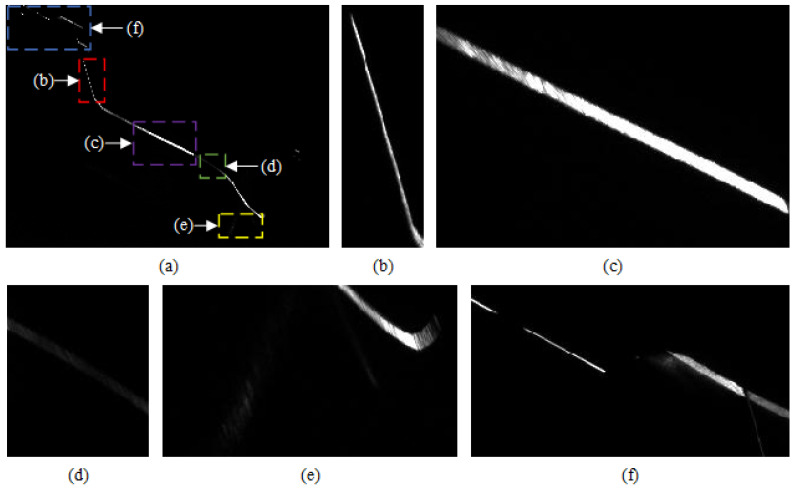
Characteristics of light stripe images from actual wheel treads. (**a**) Schematic diagram of regions corresponding to different stripe characteristics; (**b**) Narrow stripe; (**c**) Wide stripe; (**d**) Low-gray-value stripe; (**e**) Stray spots; (**f**) Stripe from other components.

The real wheel light stripe images acquired from the on-site experiment are shown in Figure 3. It can be seen that the light stripes in different regions exhibit distinct characteristics.

(1) Significant variation in light stripe width

Due to the relative positioning of the laser and camera, the width of the light stripe varies across the image, ranging from 5 to 21 pixels. Specifically, regions farther from the camera, such as the outer side of the wheel shown in Figure 3b, appear narrower, whereas regions closer to the camera, such as the tread reference point shown in Figure 3c, appear wider. These variations in stripe width, combined with non-smooth gray-level transitions and varying stripe curvature, lead to center extraction errors when using the conventional Steger method with a fixed *σ*.

(2) Uneven gray-level distribution.

When line-structured light is projected onto a tread with non-uniform surface roughness, significant differences in the gray level of the light stripe arise [36,37]. In particular, regions subjected to long-term wheel-rail contact exhibit increased reflectivity due to wear, causing the gray-level of the stripe to approach that of the background, as seen in the flange root region shown in Figure 3d. This makes it difficult for image segmentation methods based on gray-level thresholding to distinguish the stripe from background noise, further complicating center extraction.

(3) Stray spots caused by complex profile geometry.

Owing to the complex curvature of the wheel profile, stray light spots may appear along the circumferential direction in regions such as the wheel flange, as illustrated in Figure 3e. These spots interfere with accurate centerline extraction and compromise measurement accuracy.

(4) Interference from other components.

In practical applications, adjacent components such as axle boxes and brake shoes located on the outer side of the wheel can produce extraneous light stripes in the image, as shown in Figure 3f. To avoid affecting the subsequent calculation of wheel profile parameters, these irrelevant light stripes must be removed.

Given that the online measurement of geometric parameters for train wheelsets is a dynamic process, train speed fluctuates and measurement positions are inconsistent. Additionally, the impact of ambient light is variable, which further intensifies the variations in the width and gray-level distribution of the light stripes. Therefore, a targeted light stripe centerline extraction method is urgently needed. Based on the aforementioned light stripe characteristics, this paper proposes a corresponding centerline extraction method to achieve accurate, highly anti-interference light stripe centerline extraction for on-site measurement images.

## 3. Multiscale Adaptive Method for Light Stripe Center Extraction

### 3.1. Principle of the Multi-Scale Adaptive Mechanism

The standard deviation *σ* of the Gaussian convolution kernel determines the spatial scale of its smoothing effect on light stripes, and *σ* is positively correlated with the smoothing effect. In conventional laser stripe processing methods, such as the Steger algorithm, the core parameter *σ* must satisfy $\sigma \geq w/\sqrt{3}$ [38,39]. Under this condition, the second-order derivative exhibits a unique minimum within the stripe interval [−*w*, *w*], which corresponds to the stripe center. Qi et al. evaluated the statistical behavior of the Steger method in extracting the center of light stripes and concluded that the distribution of center extraction behavior in light stripe images follows a normal distribution [40]:

(1) $$N\left(\mu ,\sigma_{l}\right)=\frac{1}{\sqrt{2\pi \sigma_{l}}}\exp\left[-\frac{{\left(x-\mu \right)}^{2}}{2\sigma_{l}^{2}}\right],$$

where *μ* represents the true center of the light stripe, and *σ_l_* represents the standard deviation of the extracted center position, which can be expressed as:

(2) $$\sigma_{l}=\sqrt{\frac{{\left(\sigma^{2}+w^{2}\right)}^{3}}{8\pi \sigma^{4}w^{2}}\frac{1}{SNR}},$$

where *σ* represents the Gaussian kernel parameter, *SNR* represents the signal-to-noise ratio of a stripe image.

It can be seen from Equation (2) that the smaller the *σ_l_* corresponds to more stable the center extraction results. When considering the confidence interval, *σ_l_* can be used to characterize the accuracy of center extraction. For light stripes with different widths, a fixed *σ* yields drastically different smoothing effects. For narrow stripes, a large fixed *σ* tends to cause over-smoothing, blurring the inherent gray gradient of the strip and thereby reducing the center extraction accuracy. For stripes matching *σ*, the Gaussian kernel can effectively suppress noise without blurring the gray gradient features, resulting in the highest accuracy. For wide stripes, a small fixed *σ* fails to sufficiently suppress noise in the wide gradient region, thereby greatly reducing accuracy.

With the stripe width *w* fixed, adjusting *σ* directly modulates the balance between noise suppression and feature preservation. As *σ* increases from small to large values, the accuracy increases first and then decreases. For small *σ* values, the smoothing effect is insufficient, making gradient calculations highly sensitive to noise, increasing errors in center extraction. When *σ* is matched to *w*, the Gaussian kernel effectively suppresses noise, while retaining the stripe’s intrinsic gradient features, thereby maximizing the accuracy of center extraction. For large *σ* values, excessive smoothing can blur geometric details such as curvature and inflection points, and mix directional information from different positions, resulting in increased center extraction errors.

However, for images with variations in stripe widths, a fixed *σ* value cannot adapt to such complexity [23,26,41]. Given the aforementioned challenges, for laser stripe images with varying widths, the adoption of a fixed Gaussian kernel standard deviation *σ* inevitably degrades the center extraction accuracy; additionally, uneven grayscale distribution of the stripe itself and low contrast between the stripe and the background further exacerbate the difficulty of precise stripe segmentation. These issues collectively restrict the practical applicability of baseline methods in dynamic measurement scenarios, rendering the development of a targeted improvement scheme imperative.

The core of the multi-scale adaptive mechanism for stripe center extraction lies in the scale matching between the Gaussian kernel parameter *σ* and the stripe width *w*, a dynamic balance that achieves noise suppression and feature preservation. The proposed region-adaptive multiscale extraction method is implemented as follows:

(1) Calculate the multiscale Hessian matrix and the gray gradient features of the light stripe image.
(2) Achieve light stripe region localization and segmentation through multiscale adaptive feature enhancement.
(3) Precisely locate the sub-pixel coordinates of the light stripe through adaptive Gaussian scale selection based on maximizing the normalized multiscale eigenvalues.

### 3.2. Multiscale Feature Computation

**Figure 4:**
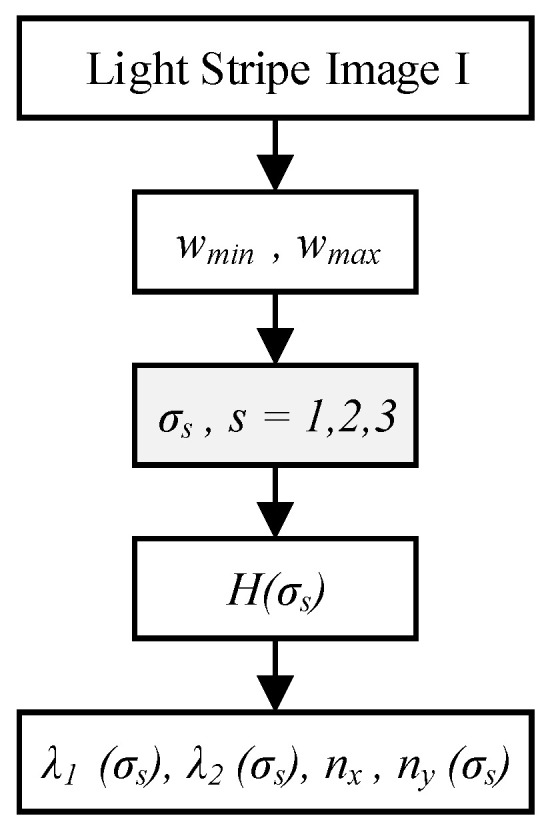
Flowchart of multiscale feature computation.

The Hessian matrix of the light stripe image I is computed using a multiscale Gaussian convolution kernel. The steps are illustrated in Figure 4:

First, based on the width range of the light stripe [*w_min_*, *w_max_*], three scale parameters *σ_s_* (*s* = 1, 2, 3) are calculated to cover the expected stripe widths. These scale parameters must satisfy the condition $\sigma \geq w/\sqrt{3}$, and are specifically defined as: $\sigma_{1}=w_{min}/\sqrt{3}$,$\sigma_{2}=\left(w_{min}+w_{max}\right)/2\sqrt{3}$, $\sigma_{3}=w_{max}/\sqrt{3}$.

Subsequently, the Hessian matrix *H*(*σ_s_*) at each scale is obtained by convolving the image *I* with the second-order derivatives of the Gaussian function *G* (*σ_s_*).

(3) $$H\left(\sigma \right)=\left[\begin{array}{cc} I_{xx} & I_{xy}\\ I_{xy} & I_{yy} \end{array}\right],$$

where *I_xx_*, *I_yy_*, and *I_xy_* represent the second-order partial derivatives of the image.

At the center of the light stripe, the eigenvalue *λ*_1_ (where |*λ*_1_| > |*λ*_2_|) of *H*(*σ*) typically reaches a minimum, reflecting a significant variation in the gray-level gradient of the stripe. Its corresponding eigenvector (*n_x_*, *n_y_*) indicates the normal direction of the stripe.

### 3.3. Multiscale Adaptive Light Stripe Enhancement and Segmentation

**Figure 5:**
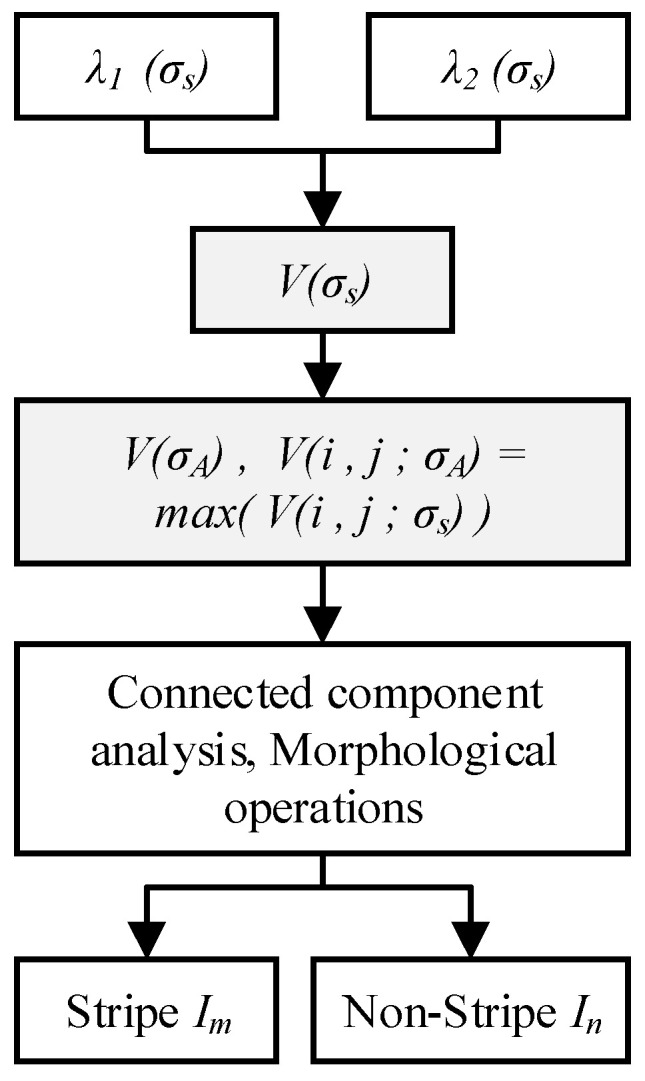
Flowchart of light stripe enhancement and segmentation.

To address the difficulty in segmenting light stripes caused by non-uniform gray-level and varying widths distribution, a feature response is constructed using multiscale Hessian eigenvalues, which not only enhances stripe features but also facilitates robust segmentation. The corresponding steps are shown in Figure 5.

**Figure 6:**
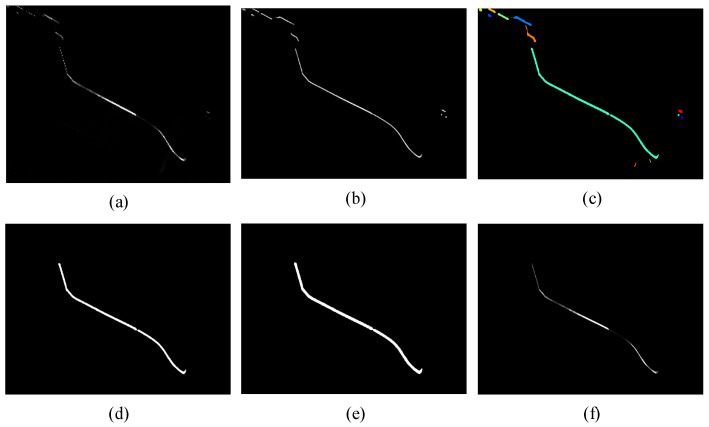
Light stripe segmentation process. (**a**) Original image; (**b**) Feature-enhanced result, where different colors represent different connected domains; (**c**) Connected components; (**d**) Stripe-connected component; (**e**) Stripe region mask; (**f**) Segmented stripe image.

First, the multiscale stripe response *V*(*σ_s_*) is calculated. Based on the multiscale eigenvalues *λ*_1_ (*σ_s_*) and *λ*_2_ (*σ_s_*), the feature response function defined in Equation (4) is constructed. This function suppresses the circularity features of the stripe according to Equation (5) and attenuates low-gray-level features according to Equation (6). Consequently, it effectively preserves stripe information and characteristics within complex image backgrounds. Specifically, it yields high response values for stripes with varying widths and gray levels while outputting low response values for background noise and stray spots, leading to effective feature enhancement [42]. The original image and the enhancement result are shown in Figure 6a and Figure 6b, respectively. It can be seen that the low-gray-level regions of the light stripe are effectively enhanced, thereby preventing the loss of stripe information during segmentation and center extraction.

(4) $$V_{\sigma }=\left\{\begin{array}{cc} 0 & \lambda_{2}\geq 0\\ \exp\left(-\frac{R_{A}^{2}}{2\beta^{2}}\right)\left(1-\exp\left(-\frac{S^{2}}{2c^{2}}\right)\right) & \lambda_{2}<0 \end{array}\right.,$$

(5) $$R_{A}=\left|\frac{\lambda_{2}}{\lambda_{1}}\right|,$$

(6) $$S=\sqrt{\lambda_{1}^{2}+\lambda_{2}^{2}},$$

where *R_A_* represents the circularity feature of the pixel, used to filter out isolated points and non-linear stray spots; *S* is employed to suppress low-gray-level background noise; *β* and *c* are adjustable parameters that regulate the sensitivity of *R_A_* and *S*, respectively.

Subsequently, an adaptive multiscale response selection is performed. For each pixel in the image, the maximum response value *max*(*V*(*i*, *j*, *σ_s_*)) across all scales is selected. This yields the maximum response map *V*(*σ_A_*), which achieves enhanced characterization of the light stripe features.

Finally, light stripe segmentation is performed. The maximum response map *V*(*σ_A_*) is converted into a binary image using a response threshold *V*th. Connected component analysis is then conducted based on pixel connectivity, with the result shown in Figure 6c. By analyzing the size and shape features of the connected components, the component corresponding to the light stripe is identified, as illustrated in Figure 6d. At this stage, the connected component containing the light stripe retains its positional information. Morphological operations are applied to fill small holes within the stripe region, forming a mask of the stripe area, as seen in Figure 6e. Finally, the light stripe region image *I_m_* is extracted using this mask, while pixels in other non-stripe regions *I_n_* are set to zero, thereby completing the segmentation, shown in Figure 6f. The segmented light stripe image is then used for subsequent center extraction tasks.

### 3.4. Multiscale Adaptive Light Stripe Center Extraction

**Figure 7:**
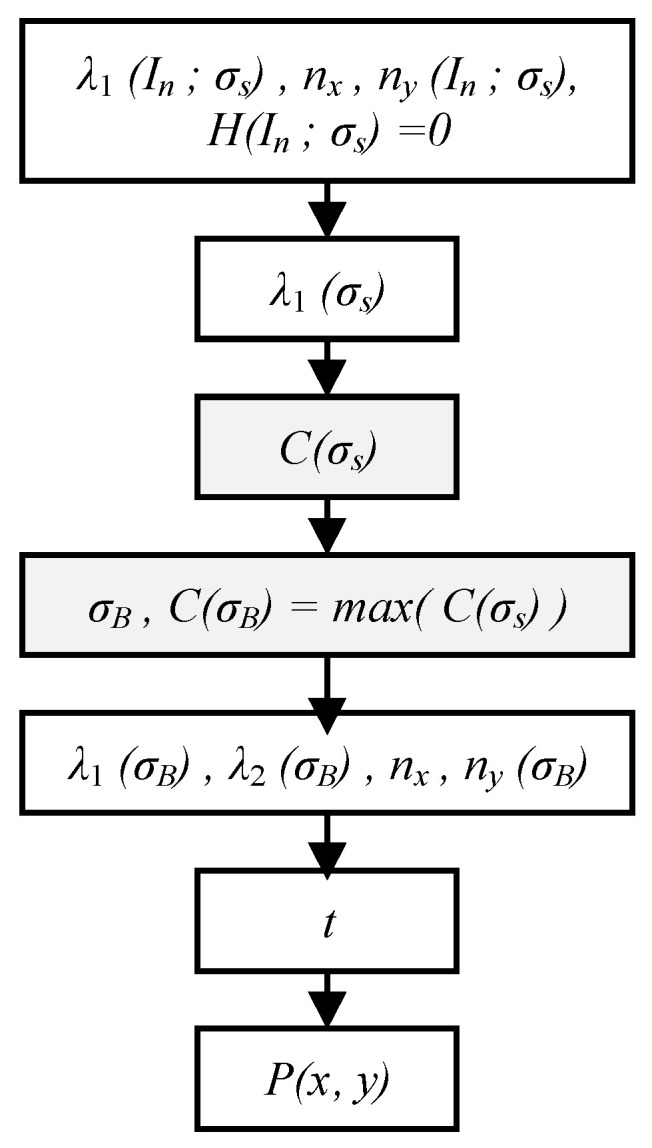
Flowchart of multiscale adaptive center extraction.

The procedure for light stripe center extraction is illustrated in Figure 7. First, the stripe features *λ*_1_, *n_x_*, *n_y_*, and *H* in the non-stripe region *I_n_* are set to zero. Next, the Gaussian kernel *σ_A_* best suited to the local stripe width is determined. Since smaller *σ* values yield larger eigenvalues *λ*_1_ within the stripe region, the eigenvalues *λ*_1_ must be normalized using Equation (7) during the adaptive multiscale feature selection [43]. The normalization operator *C*(*σ_s_*) compensates for the attenuation of the gray gradient and eigenvalues caused by larger scales, ensuring comparability of eigenvalues across different scales.

**Figure 8:**
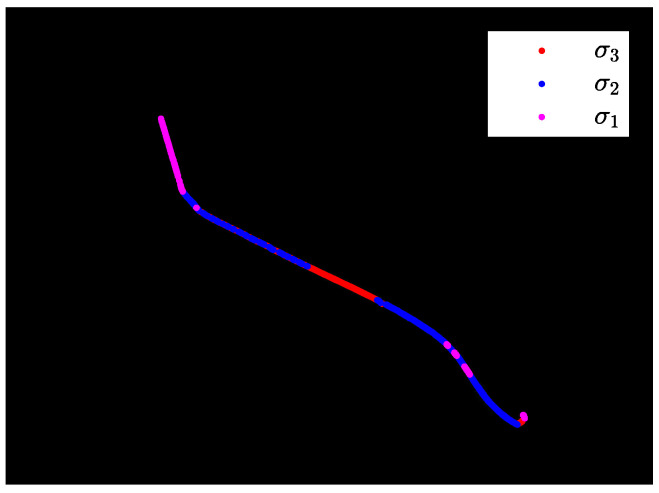
Optimal Gaussian kernel for different light stripe regions.

In this step, the scale parameter *σ_B_* corresponding to the maximum normalized operator *max*(*C*(*σ_s_*)) optimally matches the local stripe width. Figure 8 shows the optimal *σ* selected for different regions of a light stripe. This approach achieves precise adaptation to complex scenes with globally inconsistent stripe widths, effectively overcoming the limitation of the traditional Steger algorithm, where a fixed Gaussian kernel size cannot adapt to varying stripe widths, which leads to reduced center extraction accuracy.

(7) $$C(\sigma )=\sigma^{2}|\lambda_{1}|$$

Next, the sub-pixel offset of the light stripe center is calculated. A second-order Taylor expansion is performed at the initially located stripe center coordinates *P*_0_(*x*_0_, *y*_0_) using the eigenvector and the Hessian matrix corresponding to *σ_B_*. The sub-pixel offset *t* at this point is computed according to Equation (8):

(8) $$t=-\frac{n_{x}\cdot I_{x}+n_{y}\cdot I_{y}}{n_{x}^{2}\cdot I_{xx}+2\cdot n_{x}\cdot n_{y}\cdot I_{xy}+n_{y}^{2}\cdot I_{yy}},$$

where *n_x_* and *n_y_* represent the normal vector of the light stripe at the point, and *I_x_*, *I_y_*, *I_xx_*, *I_xy_*, and *I_yy_* denote the first- and second-order derivatives of the pixel’s gray value, respectively.

Finally, the sub-pixel coordinates *P*(*x*, *y*) of the light stripe center are calculated. The pixel-level coordinates are integrated with the sub-pixel offset according to Equation (9). The constraint in Equation (10) eliminates redundant information from the multiscale adaptive selection process, achieving high-accuracy light stripe coordinate extraction

(9) $$x=x_{0}+t\cdot n_{x},y=y_{0}+t\cdot n_{y},$$

(10) $$\left|t\cdot n_{x}\right|\leq 0.5,\;\left|t\cdot n_{y}\right|\leq 0.5$$

**Figure 9:**
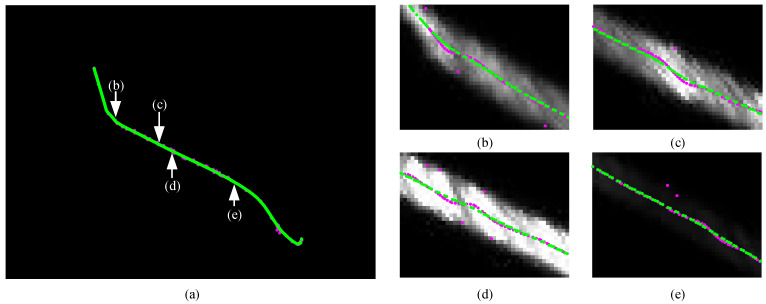
Results of light stripe center extraction for wheel profile. (**a**) Comparison of center extraction results; (**b**) Corner region; (**c**) Region with uneven gray levels; (**d**) Bright stripe region; (**e**) Dark stripe region. Green dots represent the center coordinates extracted by the proposed method, while pink dots represent those extracted by the Steger method.

The center extraction results of the proposed method and the Steger method are compared as shown in Figure 9, marked in green and purple, respectively. Subfigures (b)–(e) provide magnified views of the center extraction results. When a small *σ* is used, two symmetric minima of the second derivative may form on both sides of the stripe coordinate, resulting in spurious center points in the output. Additionally, extracting the center from stripes with non-smooth gray-level variations introduces further errors. Conversely, a large *σ* tends to smooth corner regions of the stripe into arcs, causing the extracted centerline to lose genuine geometric detail, while excessive blurring in narrow stripe regions leads to deviations in the center coordinates. The proposed method overcomes these limitations by performing region-adaptive selection of the stripe width based on multiscale maximum normalized eigenvalues, thereby improving the accuracy of center extraction.

## 4. Results and Discussion

**Figure 10:**
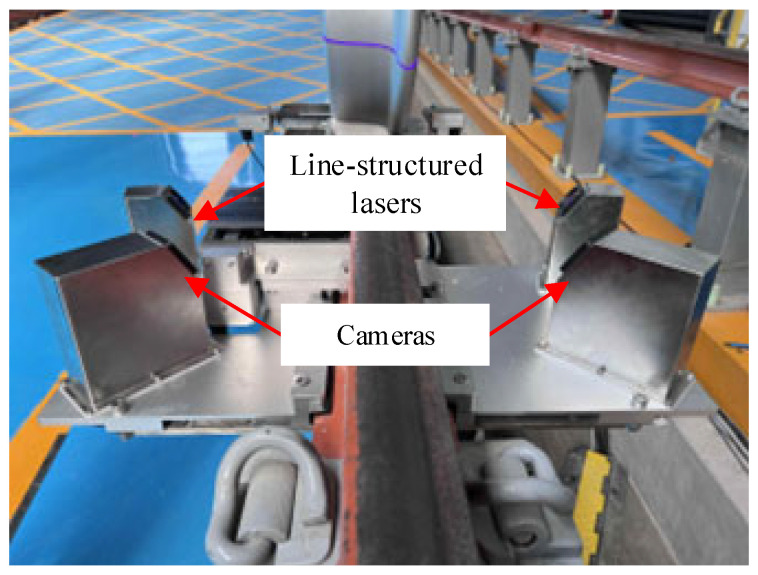
Structure of the sensor module in the wheel profile measurement system.

To validate the accuracy of the proposed method for light stripe center extraction under conditions of non-uniform surface reflectivity, experiments were conducted using the wheel profile measurement system installed at the National Railway Test Center of the China Academy of Railway Sciences. The measurement system mainly consists of an industrial personal computer (IPC) and a sensor module. The control and data processing capabilities of the system are implemented on an Advantech IPC-610L industrial personal computer, (Advantech, Kunshan, China), which is equipped with a 3.4 GHz CPU and 32 GB random access memory (RAM) to ensure stable operation of the proposed algorithm. The sensor module, as illustrated in Figure 10, comprises two line-structured lasers with a wavelength: 405 nm (line widh < 180 μm, divergence angle 45°) to project structured light onto the wheel from both sides of the rail, two Basler acA1600-60gm cameras (Basler AG, Ahrensburg, Germany) with a resolution of 1600 px × 1200 px and an exposure time set to 1200 μs, and Computar M1614-MP2 fixed-focus lenses (CBC Corporation, Tokyo, Japan) with a 16 mm focal length.

Manual measurements of the wheel’s flange height and flange thickness were conducted using a Model LLJ-4A tread profile measuring instrument (Jingyi Instrument Equipment Co., Ltd., Beijing, China) as a reference. This instrument is widely used in railway engineering, which has a division value of 0.10 mm and an inherent uncertainty of 0.03 mm. To reduce the impact of random errors on the reference value, 9 repeated measurements were performed for each wheel, and the mean value was taken as the reference value.

### 4.1. Standard Wheel Profile Measurement

First, a repeatability test of profile measurement was conducted using a standard wheel with uniform surface reflectivity. The proposed multiscale adaptive method and the conventional Steger method were applied, respectively. The results are summarized in Table 1.

The proposed method yields slightly higher mean accuracy than the traditional Steger method with a fixed *σ*. Moreover, both the standard deviation (Std) and the maximum error (ME) of the proposed method are smaller than those of the Steger method, demonstrating higher stability.

### 4.2. Real Train Wheelset Measurement

**Figure 11:**
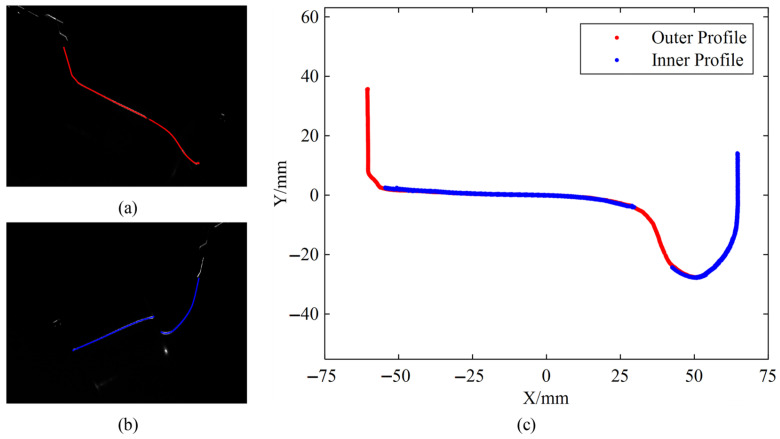
Schematic diagram of wheel profile coordinate transformation. (**a**) Outer stripe center coordinates; (**b**) Inner stripe center coordinates; (**c**) Transformed profile coordinates.

Field test data collected during actual train passes were analyzed. The train passed through the measurement system 9 times at a speed of 5 km/h. The coordinate transformation process for wheel profile measurement is illustrated in Figure 11. First, light stripe centers are extracted from the images captured by the inner and outer cameras, as shown in Figure 11a and b, respectively. Subsequently, the profile coordinates are reconstructed using calibrated parameters, including the camera extrinsic parameters and the light plane equation. The result of this coordinate transformation is displayed in Figure 11c. Finally, profile parameters are calculated based on the definitions.

Accuracy tests are performed on the same train wheel image using the proposed method and the Steger method, respectively. First, the proposed method is applied to segment the stripe image. Subsequently, both methods are adopted to extract the stripe centers from the processed image. The results are presented in Table 2. For flange height, the mean value of the measurement results obtained by the proposed method is 27.72 mm with a mean absolute error (MAE) of 0.12 mm and a Std value of 0.09 mm, thus exhibiting high precision and excellent reproducibility. In contrast, the mean measurement value of the conventional Steger method is 27.82 mm with an MAE of 0.22 mm and a Std of 0.16 mm, indicating a larger measurement deviation. For flange thickness, the proposed method achieves a mean measurement value of 32.5 mm, an MAE of 0.10 mm and a Std of 0.10 mm. However, the conventional Steger method yields a mean measurement value of 32.62 mm, an MAE of 0.22 mm and a Std of 0.22 mm. The consistent superior performance of the proposed method in both key parameters further verifies its excellent reproducibility and higher precision when processing actual complex light stripe images.

Additionally, the ME values between the flange height and flange thickness values measured by the proposed method and the manually measured reference values are 0.29 mm and 0.23 mm, respectively. These values meet the 0.4 mm accuracy requirement for dynamic wheel profile measurement in the railway industry. In contrast, the MEs of flange height and flange thickness measured by the conventional Steger method are 0.45 mm and 0.48 mm, respectively, exceeding the industry-specified accuracy threshold. This indicates that the multi-scale adaptive center extraction strategy adopted in the proposed method enhances reproducibility and stability.

To verify the reproducibility across different wheels, both the proposed method and the Steger method were used to test the images of 8 wheels. The flange height and flange thickness measurement results obtained via the two methods are presented in Table 3 and Table 4. The Std range of flange height measurements from the proposed method is 0.09–0.13 mm, while that from the conventional Steger method is 0.12–0.18 mm. For flange thickness, the Std range of the proposed method is 0.10–0.14 mm, in contrast to 0.22–0.26 mm for the conventional Steger method. It can be seen that for different wheels, the proposed method exhibits higher repeatability and reproducibility than the traditional Steger method with a fixed *σ*.

This is attributed to the uneven wear of train wheels, which induces dynamic variations in both stripe width and gray-level distribution. The conventional method adopts a fixed *σ* and is sensitive to the width of the light stripe, which ultimately affects the accuracy and integrity of light stripe extraction, resulting in deviations in measurement results. In contrast, the proposed method employs a multi-scale adaptive strategy to match the optimal *σ* for the light stripe center, which improves the stability and accuracy of center extraction.

While the region-adaptive multiscale approach effectively improves the accuracy and stability of light stripe center extraction, and its effectiveness has been validated through tests, the computational cost increases due to the multiscale Hessian matrix calculations, as well as the additional steps for stripe feature enhancement and segmentation. Tests indicate that a combined procedure using median filtering, Otsu’s method, and connected-component analysis for image denoising and stripe segmentation takes approximately 0.3 s. The conventional Steger method requires about 0.7 s for center extraction alone, yet it fails to achieve accurate stripe segmentation and stable center localization. In comparison, the proposed method spends about 1.4 s on stripe enhancement and segmentation, and an additional 0.6 s for center extraction. Consequently, the total processing time is longer than that of the traditional Steger approach.

## 5. Conclusions

To address the issue of varying light stripe widths and non-uniform gray-level distribution caused by uneven surface reflectivity in the online measurement of train wheel profiles, this paper proposes a region-adaptive multiscale method for light stripe center extraction. The core of this method lies in its multiscale adaptive mechanism, which essentially functions as a scale-matching strategy between the Gaussian kernel parameter *σ* and the stripe width *w*. By leveraging multiscale analysis to adaptively select the optimal *σ* for stripes with varying widths, this mechanism achieves a balance between noise suppression and feature preservation while enhancing the accuracy of stripe center extraction. Specifically, based on the morphological characteristics of the stripe, the method first enhances stripe features to achieve accurate segmentation. By normalizing the eigenvalues of the Hessian matrix, it adaptively determines the optimal scale parameter (*σ*) that best matches the local stripe width, thereby effectively improving the accuracy and stability of center extraction. This key multiscale adaptive capability allows the method to select the optimal scale for stripe regions of varying widths, effectively resolving the limitation of traditional fixed-kernel approaches that fail to adapt to complex stripe variations and enabling precise identification of the stripe centerline. To validate the method, centerline extraction experiments were conducted on complex stripe images acquired by a line-structured light vision sensing system for wheelset profile measurement, installed at the National Railway Test Center of the China Academy of Railway Sciences. Experimental results demonstrate that for wheels with uniform reflective surfaces, the measurement accuracy of the proposed method is marginally higher than that of the conventional Steger method. Under complex working conditions involving non-uniform wheel surface reflectivity, the proposed method achieves a measurement Std of less than 0.1 mm, a measurement bias of 0.12 mm, and a maximum measurement error below 0.3 mm, exhibiting superior repeatability and accuracy compared to the Steger method with a fixed *σ* parameter. Reproducibility tests were conducted on the light strip images of different wheels using the proposed method and Steger, respectively. The results showed that the proposed approach enables stable and reliable extraction of the light stripe center, thereby enhancing both stability and accuracy in industrial measurement environments. This research addresses the difficulty of extracting stripe centers from images affected by uneven surface roughness, a key challenge in laser vision sensing for wheelset profile measurement. Leveraging the intrinsic advantages of multiscale feature information, this multiscale adaptive approach achieves stable and accurate extraction of stripe center coordinates under complex operating conditions, providing not only an effective technical solution for high-accuracy wheelset profile measurement but also a valuable reference for similar image processing tasks in laser-based sensing. Additionally, it lays a theoretical foundation for engineering applications of such multiscale adaptive strategies.

## Figures and Tables

**Table 1:** Measurement results of the standard wheel profile (unit: mm).

Measurement Number	Conventional Steger Method		Proposed Method	
	Flange Height	Flange Thickness	Flange Height	Flange Thickness
1	27.99	32.14	28.12	32.35
2	27.99	32.11	28.03	32.16
3	28.03	32.30	27.97	32.16
4	28.19	32.46	28.04	32.23
5	28.07	32.24	28.01	32.32
6	28.05	32.42	28.03	32.26
7	27.98	32.19	28.12	32.35
8	28.22	32.44	28.03	32.25
9	28.05	32.24	28.03	32.27
10	28.18	32.38	28.02	32.16
Manual	28.00	32.20	28.00	32.20
Mean	28.08	32.29	28.04	32.25
Std	0.09	0.12	0.04	0.07
ME	0.22	0.26	0.12	0.15

**Table 2:** Measurement results of a real train wheelset. (unit: mm).

Measurement Number	Conventional Steger Method		Proposed Method	
	Flange Height	Flange Thickness	Flange Height	Flange Thickness
1	27.61	32.32	27.74	32.62
2	28.05	32.81	27.88	32.63
3	27.65	32.35	27.69	32.41
4	27.71	32.69	27.66	32.53
5	27.91	32.88	27.72	32.56
6	27.64	32.33	27.62	32.43
7	27.83	32.80	27.89	32.63
8	27.93	32.80	27.71	32.45
9	28.03	32.63	27.62	32.36
Manual	27.60	32.40	27.60	32.40
Mean	27.82	32.62	27.72	32.50
Std	0.16	0.22	0.09	0.10
ME	0.45	0.48	0.29	0.23

**Table 3:** Reproducibility measurement results of flange height. (unit: mm).

Measurement Number	Wheel #1		Wheel #2		Wheel #3		Wheel #4		Wheel #5		Wheel #6		Wheel #7		Wheel #8	
	Steger Method	Proposed Method	Steger Method	Proposed Method	Steger Method	Proposed Method	Steger Method	Pro-posed Method	Steger Method	Proposed Method	Steger Method	Proposed Method	Steger Method	Proposed Method	Steger Method	Proposed Method
1	27.61	27.74	28.07	27.63	28.01	27.65	27.85	27.85	27.94	27.88	27.92	27.70	27.75	27.88	27.81	27.53
2	28.05	27.88	27.88	27.54	27.89	27.80	27.73	27.62	27.85	27.89	27.74	27.73	27.93	27.58	27.82	27.66
3	27.65	27.69	28.07	27.87	28.01	27.85	27.64	27.89	27.71	27.71	27.52	27.63	27.56	27.59	27.82	27.59
4	27.71	27.66	27.64	27.69	27.73	27.90	28.09	27.62	27.56	27.57	27.88	27.62	28.08	27.76	27.67	27.65
5	27.91	27.72	27.91	27.56	27.79	27.53	27.67	27.66	28.04	27.55	27.54	27.77	27.70	27.75	27.76	27.85
6	27.64	27.62	27.67	27.72	27.76	27.70	27.87	27.60	28.03	27.72	27.69	27.51	27.82	27.59	27.66	27.74
7	27.83	27.89	27.90	27.58	27.61	27.67	27.66	27.62	27.66	27.69	27.82	27.54	27.87	27.87	28.03	27.60
8	27.93	27.71	27.54	27.79	27.57	27.83	27.99	27.77	27.99	27.59	27.89	27.70	27.97	27.78	27.59	27.77
9	28.03	27.62	27.92	27.59	27.85	27.84	27.65	27.71	27.86	27.89	27.92	27.55	27.99	27.67	27.77	27.64
Mean	27.82	27.72	27.84	27.66	27.80	27.75	27.79	27.70	27.85	27.72	27.77	27.64	27.85	27.72	27.77	27.67
Std	0.16	0.09	0.18	0.11	0.15	0.11	0.16	0.10	0.16	0.13	0.15	0.09	0.15	0.11	0.12	0.09

**Table 4:** Reproducibility measurement results of flange thickness (unit: mm).

Measurement Number	Wheel #1		Wheel #2		Wheel #3		Wheel #4		Wheel #5		Wheel #6		Wheel #7		Wheel #8	
	Steger Method	Proposed Method	Steger Method	Proposed Method	Steger Method	Proposed Method	Steger Method	Proposed Method	Steger Method	Proposed Method	Steger Method	Proposed Method	Steger Method	Proposed Method	Steger Method	Proposed Method
1	32.32	32.62	32.84	32.56	32.79	32.34	32.52	32.53	32.91	32.74	32.74	32.70	32.56	32.62	32.53	32.33
2	32.81	32.63	32.78	32.20	32.46	32.68	32.37	32.25	32.86	32.77	32.59	32.67	32.95	32.39	32.79	32.63
3	32.35	32.41	33.01	32.62	32.84	32.68	32.30	32.50	32.48	32.52	32.25	32.57	32.42	32.31	32.86	32.53
4	32.69	32.53	32.20	32.53	32.49	32.63	32.92	32.43	32.32	32.37	32.67	32.38	33.24	32.64	32.53	32.52
5	32.88	32.56	32.85	32.41	32.78	32.37	32.53	32.56	32.89	32.44	32.08	32.71	32.62	32.53	32.76	32.75
6	32.33	32.43	32.57	32.46	32.80	32.60	32.99	32.53	33.05	32.51	32.63	32.41	32.78	32.52	32.21	32.61
7	32.80	32.63	32.66	32.30	32.54	32.54	32.49	32.62	32.43	32.59	32.66	32.31	32.84	32.72	33.03	32.32
8	32.80	32.45	32.28	32.63	32.12	32.75	32.87	32.61	32.77	32.44	32.73	32.53	32.94	32.58	32.29	32.54
9	32.63	32.36	32.70	32.40	32.43	32.60	32.50	32.35	32.54	32.60	32.89	32.38	32.95	32.58	32.37	32.33
Mean	32.62	32.50	32.65	32.46	32.58	32.58	32.61	32.49	32.69	32.55	32.58	32.52	32.81	32.54	32.60	32.51
Std	0.22	0.10	0.25	0.14	0.23	0.13	0.24	0.12	0.24	0.13	0.24	0.14	0.23	0.12	0.26	0.14

## Data Availability

Data underlying the results presented in this paper are not publicly available at this time but may be obtained from the authors upon reasonable request.
